# Effect of antipsychotics on breast tumors by analysis of the Japanese Adverse Drug Event Report database and cell-based experiments

**DOI:** 10.1186/s40780-021-00199-7

**Published:** 2021-04-01

**Authors:** Tae Maeshima, Ryosuke Iijima, Machiko Watanabe, Satoru Yui, Fumio Itagaki

**Affiliations:** 1 Department of Clinical & Pharmaceutical Sciences, Faculty of Pharma Science, Teikyo University, 2-11-1, Kaga, Itabashi-ku, Tokyo, 173-8605 Japan; 2 Department of Medical & Pharmaceutical Sciences, Faculty of Pharma Science, Teikyo University, 2-11-1, Kaga, Itabashi-ku, Tokyo, 173-8605 Japan

**Keywords:** Breast tumor, Antipsychotics, JADER, MCF-7, Blonanserin

## Abstract

**Background:**

Since antipsychotics induce hyperprolactinemia via the dopamine D_2_ receptor, long-term administration may be a risk factor for developing breast tumors, including breast cancer. On the other hand, some antipsychotic drugs have been reported to suppress the growth of breast cancer cells in vitro. Thus, it is not clear whether the use of antipsychotics actually increases the risk of developing or exacerbating breast tumors. The purpose of this study was to clarify the effects of antipsychotic drugs on the onset and progression of breast tumors by analyzing an adverse event spontaneous reporting database and evaluating the proliferation ability of breast cancer cells.

**Methods:**

Japanese Adverse Drug Event Report database (JADER) reports from April 2004 to April 2019 were obtained from the Pharmaceuticals and Medical Devices Agency **(**PMDA) website. Reports of females only were analyzed. Adverse events included in the analysis were hyperprolactinemia and 60 breast tumor-related preferred terms. The reporting odds ratio (ROR), proportional reporting ratio (PRR), and information component (IC) were used to detect signals. Furthermore, MCF-7 cells were treated with haloperidol, risperidone, paliperidone, sulpiride, olanzapine and blonanserin, and cell proliferation was evaluated by WST-8 assay.

**Results:**

In the JADER analysis, the IC signals of hyperprolactinemia were detected with sulpiride (IC, 3.73; 95% CI: 1.81–5.65), risperidone (IC, 3.69; 95% CI: 1.71–5.61), and paliperidone (IC, 4.54; 95% CI: 2.96–6.12). However, the IC signal of breast tumors was not observed with any antipsychotics. In cell-based experiments, MCF-7 cells were treated with six antipsychotics at concentrations of 2 and 32 μM, and none of the drugs showed any growth-promoting effects on MCF-7 cells. On the other hand, blonanserin markedly suppressed the growth of MCF-7 cells at a concentration of 32 μM, and the effect was concentration dependent.

**Conclusions:**

Analysis of the JADER using the IC did not show breast tumor signals due to antipsychotic drugs. In in vitro experiments, antipsychotics did not promote MCF-7 cell proliferation whereas blonanserin suppressed MCF-7 cell growth. Further research on the effects of blonanserin on the onset and progression of breast tumor is expected.

## Background

Antipsychotics are used for the treatment of mental disorders, such as schizophrenia, and they are often taken over a long period of time. Many antipsychotics have a dopamine D_2_ receptor blocker actions, and hyperprolactinemia is a known side effect [1]. Long-term hyperprolactinemia increases the risk of developing osteoporosis and breast cancer [2, 3].

The prevalence of breast cancer is highest among cancers in women and in the leading cause of cancer death and disability-adjusted life-years for women in a 2017 global cancer morbidity and mortality report [4]. There have been several reports on the relationship between breast cancer and antipsychotics [3], and higher blood prolactin levels are associated with a higher risk of breast cancer, especially hormone receptor-positive and postmenopausal breast cancers [5]. A case-control study examining the association between antipsychotic drug use and breast cancer development found that there was no association between antipsychotic drug use and breast cancer risk but that there may be a slightly increased risk for ER-positive breast cancer [6]. However, there are reports that deny the association between breast cancer and the use of antipsychotics [7, 8], and no conclusions on this matter have been reached.

On the other hand, some antipsychotics have been confirmed in vitro to have growth inhibitory effects on breast cancer cells. For example, it was reported that pimozide and haloperidol had growth inhibitory effects on MCF-7 cells, whereas clozapine did not affect growth [9, 10]. It has also been reported that aripiprazole, which is an atypical antipsychotic, suppresses the growth of breast cancer cells such as MCF-7 cells and is comparable to an antineoplastic drug [11].

Therefore, we first analyzed the use of antipsychotics and the onset of breast tumors, including breast cancer, using the Japanese Adverse Drug Event Report database (JADER). The JADER is a Japanese drug side effect database managed by PMDA. Database information have been collected since 2004 and published since 2012. Qualitative evaluation by signal detection while using such a database serves as a guide for screening adverse events (AEs) specific to a drug. However, there are no reports on the risk of developing breast tumors in the JADER analysis. We analyzed not only breast cancer but also hyperprolactinemia, which is considered a risk factor for breast cancer.

Next, to clarify the direct effects of antipsychotics on the growth of breast cancer cells, we treated MCF-7 cells with antipsychotics that were shown to have the potential to influence the development of breast tumor based on JADER analysis and assessed cell proliferation.

## Materials and methods

### Signal detection in JADER

JADER AE reports were obtained from the PMDA website (https://www.pmda.go.jp/safety/info-services/drugs/adr-info/suspected-adr/0003.html). The database consists of four datasets in csv format: patient demographic information (DEMO), drug information (DRUG), AE information (REAC), and primary disease information (HIST). These tables can be connected using the case ID number. We analyzed data from April 2004 to April 2019. The analysis was performed on data only from females. In the DRUG table, the item “participation of drug” included only “suspected drugs.” The names of the AEs used in the JADER are standardized in the preferred term (PT) of the Medical Dictionary for Regulatory Activities (MedDRA). The AEs of breast tumors were identified by PTs included in the MedDRA defined “HLT: benign and malignant breast neoplasms” (Table 1). They were coded according to MedDRA version 22.0.

**Table 1:** Preferred terms included in the “benign and malignant breast neoplasms” used in this study

PT	code
Benign breast neoplasm	10004243
Breast cancer	10006187
Breast cancer in situ	10006189
Breast cancer recurrent	10006198
Breast cancer stage I	10006199
Breast cancer stage II	10006200
Breast cancer stage III	10006201
Breast cancer stage IV	10006202
Breast cyst	10006220
Breast hyperplasia	10006256
Breast neoplasm	10006279
Fibroadenoma of breast	10016613
Fibrocystic breast disease	10016621
Haemangioma of breast	10018820
Inflammatory carcinoma of breast recurrent	10021977
Inflammatory carcinoma of breast stage III	10021978
Inflammatory carcinoma of breast stage IV	10021979
Inflammatory carcinoma of the breast	10021980
Intraductal papilloma of breast	10022781
Lipoma of breast	10024616
Medullary carcinoma of breast	10027095
Metastases to breast	10027454
Paget’s disease of nipple	10033364
Malignant nipple neoplasm male	10053128
Malignant nipple neoplasm female	10053129
Breast cancer metastatic	10055113
Nipple neoplasm	10056286
Breast cancer female	10057654
Breast cancer male	10061020
Malignant nipple neoplasm	10062051
Breast adenoma	10063087
Breast fibroma	10063088
HER2 positive breast cancer	10065430
Apocrine breast carcinoma	10066206
Breast sarcoma	10068582
Breast sarcoma metastatic	10068583
Breast sarcoma recurrent	10068584
Oestrogen receptor positive breast cancer	10070577
Phyllodes tumour	10071776
Benign nipple neoplasm	10072040
Breast cyst rupture	10072812
Breast angiosarcoma	10072813
Breast angiosarcoma metastatic	10072814
Intraductal proliferative breast lesion	10073094
Invasive ductal breast carcinoma	10073095
Invasive lobular breast carcinoma	10073096
Invasive papillary breast carcinoma	10073098
Lobular breast carcinoma in situ	10073099
Metaplastic breast carcinoma	10073100
Mucinous breast carcinoma	10073101
Neuroendocrine breast tumour	10073103
Tubular breast carcinoma	10073104
Intraductal papillary breast neoplasm	10073540
Triple negative breast cancer	10075566
Invasive breast carcinoma	10075713
Hormone refractory breast cancer	10076935
Haemorrhagic breast cyst	10077443
Intracystic breast papilloma	10078162
Squamous cell breast carcinoma	10079307
Primary breast lymphoma	10081036

The four tables constituting the JADER were subjected to deduplication and data combination using JMP pro 14.1.0 (SAS Institute Inc., Cary, NC, US). ROR, PRR, and IC were used for signal detection. The ROR was based on a paper by Kenneth J Rothman et al. [12], and a signal was present when the lower limit of the 95% CI exceeded 1. The PRR was based on a paper by S J Evans et al. [13] and a signal was present when the following criteria were met: PRRs ≥2, χ^2^ ≥ 4, and *n* ≥ 3. The IC was based on a paper by A Bate et al. [14], and a signal was present when the lower limit of the 95% CI exceeded 0.

The antipsychotic drugs analyzed in this study are N05A in the WHO ATC classification, and there are 29 drugs of the group marketed in Japan as of August 2019.

### Effects of antipsychotics on breast cancer cell proliferation

#### Materials

Haloperidol, sulpiride, risperidone, paliperidone, blonanserin and olanzapine were purchased from Tokyo Chemical Industry (Tokyo Japan). Dimethyl sulfoxide (DMSO) and tamoxifen citrate were purchased from FUJIFILM Wako Pure Chemical Corporation (Osaka, JAPAN). All drugs were dissolved in DMSO to a final DMSO concentration of 0.5%. The Cell Counting Kit-8 was purchased from Dojindo Molecular Technologies (Kumamoto, Japan).

#### Cell culture

MCF-7 cells (ATCC, Manassas, VA) were cultured in RPMI-1640 medium (SIGMA-Aldrich, MO, USA) containing 10% FBS (BIOWEST, France), 80 μg/mL kanamycin (SIGMA-Aldrich, MO, USA), and 0.3 mg/mL L-glutamine (SIGMA-Aldrich, MO, USA) at 37 °C with 5% CO_2_ confluent cultures passaged by trypsinization (FUJIFILM Wako Pure Chemical Corporation, Osaka, JAPAN).

#### WST-8 assay

MCF-7 cells (2.5 × 10^3^/well) were seeded in 96-well plates and incubated for 24 h. Then, the cells were treated with different concentrations of antipsychotics and tamoxifen citrate. The medium was exchanged 48 h after seeding the cells. After treatment for 72 h, the cells were washed with PBS. Then, 100 μL 10% FBS-RPMI+ 10 μL WST-8 solution was added to each well and incubated for an additional 1 h at 37 °C with 5% CO_2_. Absorbance at 450 nm was measured with a microplate reader (DS Pharma Biomedical, Osaka, Japan). All assays were replicated three times.

#### Statistical analysis

JMP pro14.1.0 was used for statistical analysis. The results are shown as the mean ± S.E. or S.D. Data were analyzed by one-way analysis of variance combined with Dunnett’s test or Tukey’s test. *P* < 0.05 was considered to indicate a statistically significant difference.

## Results

### JADER analysis

The total number of JADER cases reported was 6,339,117, and 703,846 cases of data from women with duplicate data deleted were analyzed. The analysis results are shown in Tables 2 and 3. Among the conditions reports for 29 antipsychotics, “hyperprolactinemia” was reported for 9 antipsychotics, and “benign and malignant breast neoplasms” were reported for 10 antipsychotics. There were six antipsychotics, namely, haloperidol, paliperidone, risperidone, sulpiride, olanzapine, and blonanserin, for which both “hyperprolactinemia” and “benign and malignant breast neoplasms” were reported.

**Table 2:** Signal scores for antipsychotics-associated “Benign and malignant breast neoplasms”

		total ^a)^	Benign and malignant breast neoplasms			
			n	ROR (95%CI)	PRR (X^2^)	IC (95%CI)
Typical antipsychotic						
	Haloperidol	939	1	0.49 (0.07, 3.51)	0.49 (0.13)	−0.60 (−5.30, 4.11)
	Bromperidol	113	1	4.14 (0.58, 29.68)	4.11 (0.27)	0.58 (−4.06, 5.43)
	Pipamperone	15	0	–	–	–
	Spiperone	0	0	–	–	–
	Timiperone	9	0	–	–	–
	Propericiazine	85	0	–	–	–
	Prochlorperazine	143	0	–	–	–
	Perphenazine	17	0	–	–	–
	Fluphenazine	89	0	–	–	–
	Levomepromazine	730	0	–	–	–
	Chlorpromazine	431	0	–	–	–
	Sulpiride	1395	12	4.05 (2.29, 7.16)*	4.02 (24.15)*	1.70 (−0.16, 3.56)
	Sultopride	52	0	–	–	–
	Nemonapride	9	0	–	–	–
	Pimozide	39	0	–	–	–
Atypical antipsychotic						
	Perospirone	416	0	–	–	–
	Risperidone	2629	11	1.95 (1.08, 3.54)*	1.95 (4.17)	0.84 (−1.08, 2.78)
	Paliperidone	774	2	2.37 (0.59, 9.53)	2.37 (0.51)	0.70 (−3.16, 4.55)
	Blonanserin	550	4	3.4 (1.27, 9.11)*	3.39 (4.54)*	1.19 (−1.80, 4.19)
	Olanzapine	1825	2	0.51 (0.07, 3.51)	0.5 (0.52)	−0.72 (−4.56, 3.13)
	Quetiapine	1836	0	–	–	
	Clozapine	1283	11	4.03 (2.22, 7.31)*	4.01 (21.77)*	1.67 (−0.26, 3.61)
	Asenapine	134	0	–	–	–
	Aripiprazole	2316	6	1.36 (0.61, 3.03)	1.36 (0.26)	0.37 (−2.15, 2.89)
	Brexpiprazole	112	0	–	–	–
	Zotepine	279	5	8.48 (3.50, 20.58)*	8.35 (25.39)*	1.94 (−0.84, 4.65)
	Mosapramine	4	0	–	–	–
	Oxypertine	6	0	–	–	–
	Clocapramine	0	0	–	–	–

ROR: the reporting odds ratio, PRR: the proportional reporting ratio, IC: the information component, CI: the confidence interval

a) total number of reports from April 2004 to April 2019 in JADER

An asterisk(*) indicates that the adverse events are detected as signals

**Table 3:** Signal scores for antipsychotics-associated “Hyperprolactinemia”

		total ^a)^	Hyperprolactinemia			
			n	ROR (95%CI)	PRR (X^2^)	IC (95%CI)
Typical antipsychotic						
	Haloperidol	939	3	81.79 (25.76, 259.68)*	80.89 (158.65)*	1.95 (−1.47, 5.36)
	Bromperidol	113	0	–	–	–
	Pipamperone	15	0	–	–	–
	Spiperone	0	0	–	–	–
	Timiperone	9	0	–	–	–
	Propericiazine	85	0	–	–	–
	Prochlorperazine	143	0	–	–	–
	Perphenazine	17	0	–	–	–
	Fluphenazine	89	4	149.56 (54.48, 410.63)*	146.59 (424.31)*	2.28 (−0.80, 5.36)
	Levomepromazine	730	0	–	–	–
	Chlorpromazine	431	0	–	–	–
	Sulpiride	1395	13	283.29 (156.78, 511.89)*	273.74 (2840.96)*	3.73 (1.81, 5.65)*
	Sultopride	52	0	–	–	–
	Nemonapride	9	0	–	–	–
	Pimozide	39	0	–	–	–
Atypical antipsychotic						
	Perospirone	416	0	–		
	Risperidone	2629	13	190.01 (105.46–342.34)*	185.67 (1919.94)*	3.69 (1.78, 5.61)*
	Paliperidone	774	23	1187.33 (728.48,1935.18)*	1050.90 (17,778.71)*	4.54 (2.96, 6.12)*
	Blonanserin	550	5	184.22 (74.16, 457.61)*	179.78 (683.21)*	2.54 (−0.29, 5.37)
	Olanzapine	1825	4	60.79 (22.27, 165.95)*	60.30 (170.62)*	2.22 (−0.83, 5.28)
	Quetiapine	1836	1	15.24 (2.12,109.53)*	15.21 (2.84)	0.91 (−3.85, 5.66)
	Clozapine	1283	0	–	–	–
	Asenapine	134	2	188.96 (45.76, 780.12)*	184.14 (200.09)	1.57 (−2.39, 5.53)
	Aripiprazole	2316	0	–	–	–
	Brexpiprazole	112	0	–	–	–
	Zotepine	279	0	–	–	–
	Mosapramine	4	0	–	–	–
	Oxypertine	6	0	–	–	–
	Clocapramine	0	0	–	–	–

ROR: the reporting odds ratio, PRR: the proportional reporting ratio, IC: the information component, CI: the confidence interval

a) total number of reports from April 2004 to April 2019 in JADER

An asterisk(*) indicates that the adverse events are detected as signals

Analysis of hyperprolactinemia using ROR detected signals with nine drugs: haloperidol, fluphenazine, sulpiride, risperidone, paliperidone, blonanserin, olanzapine, quetiapine and asenapine. In the analysis using PRR, signals were detected with seven drugs: haloperidol, fluphenazine, sulpiride, risperidone, paliperidone, blonanserin, and olanzapine. Signals were detected with three drugs, namely, sulpiride, risperidone, and paliperidone, by IC analysis. Analysis of benign and malignant breast neoplasms was done using ROR detected signals with five drugs: sulpiride, risperidone, blonanserin, clozapine, and zotepine. In the analysis using PRR, signals were detected with four drugs: sulpiride, blonanserin, clozapine, and zotepine. None of the drugs had signals detected using IC.

### Effect of antipsychotic drugs on proliferation of MCF-7 cells

**Fig. 1:**
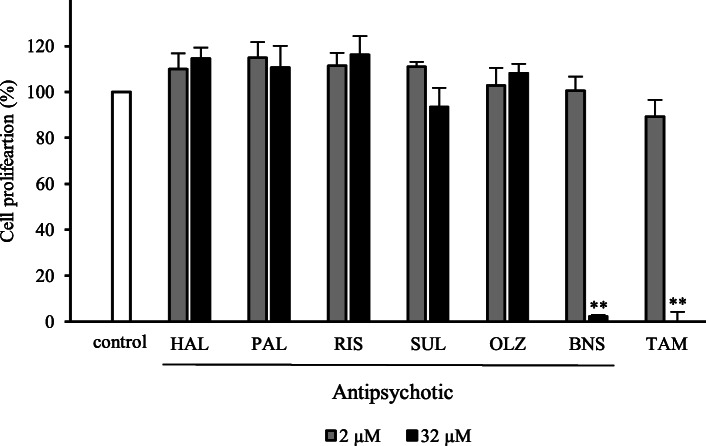
Effect of Antipsychotic on the proliferation of MCF-7 cells as assessed by WST-8 assay. TAM, which is known to suppress the growth of MCF-7 cells, was also exposed. Values are the mean ± S.E.(n = 4). **p < 0.01 compared with control. HAL: Haloperidol, PAL: Paliperidone, RIS: Risperidone, SUL: Sulpiride, OLZ: Olanzapine, BNS: Blonanserin

**Fig. 2:**
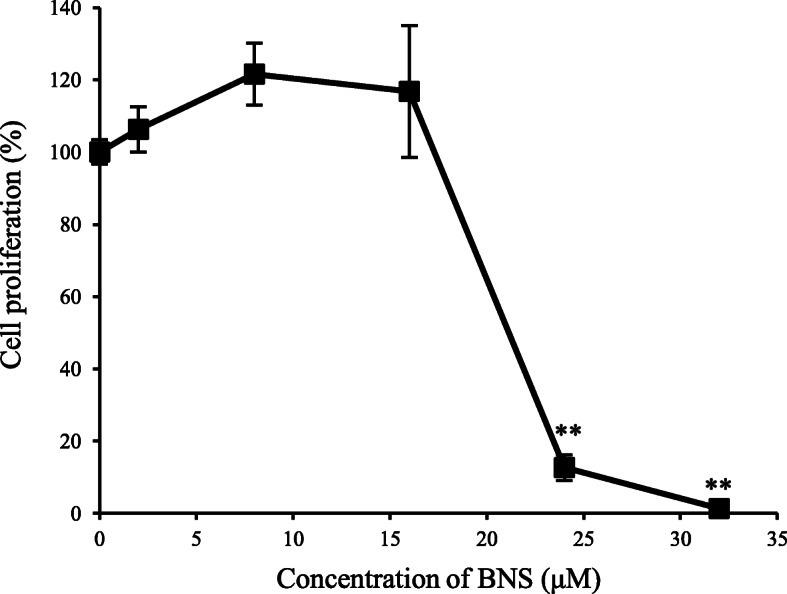
Effect of Blonanserin on the proliferation of MCF-7 cells as assessed by WST-8 assay. Values are the mean ± S.E.(*n* = 4). **p < 0.01 compared with control. BNS: Blonanserin

We used six antipsychotics for cell experiments, both of which were reported as “hyperprolactinemia” and “benign and malignant breast neoplasms” in the JADER. When the antipsychotics haloperidol, paliperidone, risperidone, sulpiride, olanzapine and blonanserin were added to MCF-7 cells at 2 and 32 μM, none of the drugs showed any effect of promoting the growth of MCF-7 cells. Conversely, MCF-7 cells exposed to 32 μM blonanserin had markedly suppressed proliferation (Fig. 1). Furthermore, when the effect of 2–32 μM blonanserin on the proliferation of MCF-7 cells was evaluated, proliferation was significantly suppressed at 24 μM or higher (Fig. 2).

## Discussion

In the JADER analysis of antipsychotics related to the AEs of breast tumor, a signal was detected with five drugs using ROR and four drugs using PRR, and no signal was detected using IC. There are several methods for detecting signals in the AE spontaneous reporting database, and ROR, PRR, and IC were used in this study. It is known that the commonly used ROR and PRR are similar when the incidence of AEs is low. In addition, it has been noted that there are many false positives because of the inflation of the values ​​due to random fluctuations [15]. On the other hand, the Bayesian method, IC, is designed to avoid inflation even in the event of few reports. Since there were few reports of AEs related to hyperprolactinemia and breast tumors due to the antipsychotics analyzed in this study, we focused on the results using IC. Our analysis study was based on the number of drugs and not on the number of cases. Therefore, it includes both single agents and combinations of agents.

Sulpiride, risperidone, and paliperidone, which had signals for hyperprolactinemia detected by IC analysis, were not detected in benign and malignant breast neoplasms. Although hyperprolactinemia due to antipsychotics is already known [1], a meta-analysis reported that patients with hyperprolactinemia did not have an increased risk of breast cancer [16]. The analyzed results of this study supported this paper. We also calculated chlorpromazine equivalents for the three drugs of sulpiride, risperidone, and Paliperidone. However, discussions were challenging because of the large number of missing values.

Typical, rather than atypical, antipsychotics are generally thought to cause hyperprolactinemia. Additionally, it has been reported that the higher the pituitary D2 receptor occupancy is, that is, the more difficult it is to cross the BBB, the higher the risk of hyperprolactinemia will be [17]. However, in the JADER used in this study, the total number of reports for atypical antipsychotics exceeds that for typical antipsychotics. In addition, there are large variations in the total number of reports due to differences in the amount of each antipsychotic drug used. Spontaneous reporting data, including JADER, contains subjective assessments, hence, duplicate reporting, underreporting, and reporting bias can affect analysis results. In addition, the date lacked detailed individual information. These are the limitations of the research. In addition to antipsychotics, H_2_ blockers, tricyclic antidepressants, SSRIs, antihypertensives, estrogen therapy, and opioids [18–22] among others, are known factors in drug-induced hyperprolactinemia. Therefore, concomitant medications may also have an impact in individual cases. In this study, there were cases in which fluphenazine and duloxetine were used in combination, and cases in which risperidone and sulpiride were used in combination with domperidone. However, it is difficult to comment of its impact on the results because details, such as the timing of combined use, are unknown, and the possibility of duplicate reporting cannot be ruled out.

We also confirmed whether the subjects had any disease that affects hyperprolactinemia.

Of the 68 cases of hyperprolactinemia who used antipsychotics, one had a pituitary tumor and the other had hypothyroidism. There were no other reports, such as polycystic ovary syndrome, that were shown to be associated with hyperprolactinemia.

In experiments with breast cancer cells, we confirmed the direct effects of antipsychotic drugs on cell proliferation. Since 2/3 of breast cancers are positive for estrogen receptors [23], we used ER(+) MCF-7 cells in our experiments. It has been reported that MCF-7 cells also express dopamine, serotonin acetylcholine receptors and sigma receptors [24–27]. Haloperidol, paliperidone, risperidone, sulpiride, and olanzapine showed no effects in the evaluation of cell proliferation 72 h after MCF-7 cell exposure to antipsychotics. Haloperidol has been reported to suppress MCF-7 cell proliferation which suggests that its action is sigma receptor mediated [9, 10]., but its effect could not be confirmed in our study. This may be due to differences in protocols such as culture conditions. Sulpiride, which has a D_2_ receptor blockade, does not act on MCF-7 cells by itself, but it has been shown to increase responsiveness to dexamethasone [28]. Our experiments also showed that sulpiride had no effect on the proliferation of MCF-7 cells. Phenothiazines have also been shown to inhibit the PDK1/Akt pathway, which plays an important role in cell survival, proliferation, and tumorigenesis [29]. On the other hand, bromocriptine, which has a dopamine receptor agonistic effect, has also been shown to induce apoptosis via the D_2_ receptor and inhibit MCF-7 proliferation [30]. An antipsychotic drug that induces hyperprolactinemia has been shown to promote the progression of precancerous cells to cancerous cells via JAK/STAT5 [31]. Increased expression levels of DRD2 mRNA have been reported in breast cancer patients [32], so it is expected that the risk of breast cancer with antipsychotics and the actions and mechanisms mediated by dopamine receptors will be further elucidated.

Blonanserin was an antipsychotic drug launched in Japan in 2008 and is used in only some Asian countries. The blonanserin patch was launched in 2019, and it is expected that the number of patients using it will increase. Blonanserin, unlike most atypical antipsychotics, has a higher affinity for dopamine D_2_ than the serotonin 5-HT_2A_ receptor [33]. In cultured cells and in animal studies, D_2_ receptor antagonists have antitumor effects [29, 34], and blonanserin, which has a higher affinity for D_2_ receptors than other atypical antipsychotics, may have a significant inhibitory effect on MCF-7 cell proliferation. The incidence of hyperprolactinemia has been reported to be low in patients who continue to receive blonanserin [35]. In our analysis, there was no IC signal for blonanserin in hyperprolactinemia. The effect of blonanserin on breast cancer cells was confirmed in SUM159, a triple-negative breast cancer cell line used often in brain metastasis studies, with an IC50 of > 100 μM [36]. Our results are the first report to clarify the effect of blonanserin on the proliferation of MCF-7 cells. In this study, a significant growth inhibitory effect was observed at 24 μM, so the effect may differ depending on the cell type. The clinical significance of this effect is not yet clear. The maximum blood concentration of blonanserin in humans is approximately 2 nM, which is in the nM rather than μM range. In addition, the effects of other antipsychotics on MCF-7 were confirmed at concentrations equal to or higher than blood levels. Other antipsychotics also showed antiproliferative effects on MCF-7 cells at similar concentrations, and there may also be class effects.

Although not shown in this paper, we predicted the binding ability of each antipsychotic drug to the estrogen receptor using the ADMET Predictor™ software, which can predict the binding ability to the estrogen receptor from a structure-activity relationship. Among the six antipsychotics used in this study, blonanserin and its major metabolites showed the highest predicted value to bind to the estrogen receptor. If the inhibitory effect of blonanserin on the growth of MCF-7 cells is mediated by the estrogen receptor, there is a possibility that such a difference in binding ability may have an influence. In addition, although our software cannot predict the ability of blonanserin to bind to the prolactin receptor, it has been reported that diphenylbutylpiperidine antipsychotics bind to the prolactin receptor and suppress signal transduction [37]. Therefore, other antipsychotics may act via the prolactin receptor.

This is the first report to evaluate the association between antipsychotics and the development of breast cancer using the JADER. Furthermore, we confirmed the effects of antipsychotic drugs, which were reported in the JADER for hyperprolactinemia and breast cancer, on the proliferation of MCF-7 cells. Since typical antipsychotics and atypical antipsychotics simply cannot be compared, it is necessary to select the appropriate drug for each patient [38]. Various factors are related to the influence of antipsychotic drugs in terms of the occurrence and progression of breast cancer, and this may be a consideration in choosing the remedy that is least likely to cause hyperprolactinemia and that has a suppressive effect on the growth of breast cancer cells from the viewpoint of side effects.

## Conclusions

Analysis of the JADER using IC did not reveal signals for the development of breast tumors due to antipsychotic drugs. In the in vitro experiments, MCF-7 cell growth was not promoted by haloperidol, paliperidone, risperidone, sulpiride, olanzapine, or blonanserin; however, blonanserin was observed to suppress MCF-7 cell growth.

## Data Availability

The dataset acquired and analyzed in this study will be made available to the responsible authors upon due request.

## Abbreviations

JADER: Japanese Adverse Drug Event Report database
PMDA: Pharmaceuticals and Medical Devices Agency
AE: adverse event
MCF-7: Michigan Cancer Foundation-**7**
ROR: reporting odds ratio
PRR: proportional reporting ratio
IC: information component
ER: estrogen receptor
CI: confidence interval
DMSO: dimethyl sulfoxide
FBS: fetal bovine serum
RPMI: Roswell Park Memorial Institute media
TAM: tamoxifen
PBS: phosphate-buffered saline
SSRI: selective serotonin reuptake inhibitor
PDK: phosphoinositide-dependent kinase
DRD: dopamine receptor gene
WHO: World Health Organization
ATC: Anatomical Therapeutic Chemical
